# Signaling Overlap between the Golgi Stress Response and Cysteine Metabolism in Huntington’s Disease

**DOI:** 10.3390/antiox10091468

**Published:** 2021-09-15

**Authors:** Bindu D. Paul

**Affiliations:** 1 Department of Pharmacology and Molecular Sciences, Johns Hopkins University School of Medicine, Baltimore, MD 21205, USA; bpaul8@jhmi.edu; 2 The Solomon H. Snyder Department of Neuroscience, Johns Hopkins University School of Medicine, Baltimore, MD 21205, USA; 3 Department of Psychiatry and Behavioral Sciences, Johns Hopkins University School of Medicine, Baltimore, MD 21205, USA

**Keywords:** Golgi apparatus, Huntington’s disease, cysteine, transsulfuration, Golgi stress response, integrated stress response

## Abstract

Huntington’s disease (HD) is caused by expansion of polyglutamine repeats in the protein huntingtin, which affects the corpus striatum of the brain. The polyglutamine repeats in mutant huntingtin cause its aggregation and elicit toxicity by affecting several cellular processes, which include dysregulated organellar stress responses. The Golgi apparatus not only plays key roles in the transport, processing, and targeting of proteins, but also functions as a sensor of stress, signaling through the Golgi stress response. Unlike the endoplasmic reticulum (ER) stress response, the Golgi stress response is relatively unexplored. This review focuses on the molecular mechanisms underlying the Golgi stress response and its intersection with cysteine metabolism in HD.

## 1. Introduction

Huntington’s disease (HD) is an autosomal dominant neurodegenerative disease which profoundly affects the corpus striatum of the brain; it results from expansion of polyglutamine repeats in the protein huntingtin [1]. Mutant huntingtin (mHtt) aggregates and affects cellular processes in multiple ways [2]. mHtt affects basic neuronal processes such as transcription, translation, nuclear-cytoplasmic transport, redox homeostasis, mitochondrial function and amino acid metabolism in addition to a myriad of physiological processes [3,4,5,6,7].

**Figure 1:**
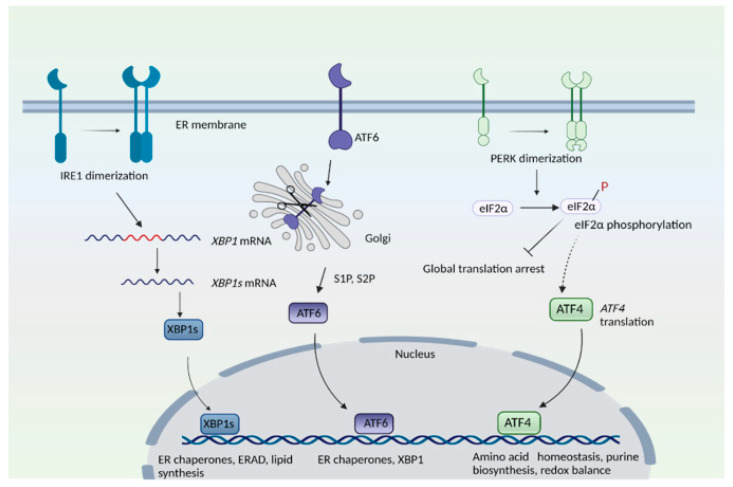
The endoplasmic reticulum (ER) stress response. The mammalian ER stress response consists of three arms: the inositol-requiring enzyme 1 (IRE1), protein kinase R (PKR)-like ER kinase (PERK), and activating transcription factor 6 (ATF6) pathways. IRE1 senses ER stress, which leads to its dimerization and to the activation of its endonuclease role, that is, to splice a specific intron from the mRNA of X-box binding protein 1, XBP1 to create XBP1s. T XBP1s protein translocates to the nucleus and transactivates its target genes. In the ATF6 arm, binding immunoglobulin protein/glucose-regulated protein 78 (BiP/GRP78) dissociates from ATF6 when unfolded proteins accumulate in the ER. ATF6 translocates to the Golgi complex, where it undergoes proteolytic cleavage by site 1 and site 2 proteases (S1P and S2P). The N-terminal cytosolic fragment of ATF6 migrates to the nucleus and induces expression of target genes. In the PERK arm, dissociation of BiP causes its dimerization and autophosphorylation. PERK then phosphorylates eukaryotic initiation factor 2α (eIF2α), which results in global translational arrest. Under these conditions, only certain mRNAs such as *ATF4* are translated, in order to maintain cellular functions during stress.

HD has also been linked to impaired stress responses involving redox homeostasis and endoplasmic stress response [5,7]. In addition to essential functions in cellular function, organelles serve important roles as sensors of stress and as hubs for signaling pathways. For instance, the endoplasmic reticulum (ER) plays central roles in protein folding, post-translational modifications, quality control of proteins and Ca^2+^ handling, among many other functions [8,9,10,11]. During ER stress—a state of functional imbalance—adaptive and restorative programs such as the unfolded protein response (UPR) and ER-associated protein degradation (ERAD), or autophagy, come into play [12,13]. One stimulus that triggers the ER stress response is the accumulation of unfolded or misfolded proteins in the ER lumen. Three arms exist in the ER stress response: the protein kinase R (PKR)-like endoplasmic reticulum kinase (PERK), activating transcription factor 6 (ATF6), and inositol-requiring enzyme 1 (IRE1) pathways, where each of the sensor proteins is a membrane protein (Figure 1). In the PERK arm, during stress, PERK dissociates from the chaperone protein, binding immunoglobulin protein/glucose-regulated protein 78 (BiP/GRP78), and undergoes dimerization and phosphorylation. PERK, (a component of the integrated stress response) then phosphorylates the eukaryotic translation initiation factor 2 subunit −α (eIF2α), which results in global translational arrest. Under these conditions only certain mRNAs such as those encoding activating transcription factor 4 (ATF4) are translated, in order to maintain functions important for cell survival. ATF4 regulates amino acid homeostasis, purine metabolism, response to oxidative stress, autophagy and apoptosis.

In the inositol-requiring enzyme (IRE) branch, the ER-resident IRE1 senses unfolded proteins or lipid disequilibrium and undergoes dimerization and autophosphorylation, activating IRE1′s cytosolic endonuclease domain, which then splices a specific intron from the mRNA of X-box binding protein 1u, *XBP1u* to create *XBP1s*. The XBP1s protein translocates to the nucleus and transactivates genes involved in protein degradation, protein folding, and lipid metabolism [14,15]. The third arm of the UPR consists of ATF6, an ER transmembrane protein that translocates to the Golgi when activated. During ER stress, when unfolded proteins accumulate, BiP/GRP78 dissociates from ATF6 to cause translocation of ATF6 into the Golgi. In the Golgi, site 1 protease (S1P) and site 2 protease (S2P) cleave ATF6 [16]. The N-terminal region of ATF6 functions as a transcription factor and stimulates expression of target genes, such as protein disulfide isomerase (PDI), XBP1, and C/EBP Homologous Protein (CHOP) [16,17]. When proteins cannot be repaired or folded back into their functional configurations, they are targeted for degradation by ERAD [18]. When the repair capacity of ERAD is crossed, portions of the ER can be specifically targeted for degradation through autophagy (ER-phagy) [19]. Recently, we elucidated the involvement of signaling pathways modulated by the Golgi apparatus in HD [20,21]. This review focuses on the involvement of the Golgi and stress signaling coordinated by this organelle in neurodegenerative states.

## 2. The Golgi Apparatus

### 2.1. Organization of the Golgi Apparatus

The Italian anatomist Camillo Golgi was the first to describe the Golgi apparatus in 1898 [22]. He developed the staining protocol for the Golgi, termed the ‘Black Reaction’ (La reazione nera) or Golgi’s staining, which accelerated the study of the brain by facilitating the microscopic visualization of the complexity of the human nervous system [23,24,25]. The Golgi apparatus both processes and sorts lipids and proteins through the secretory pathway. The Golgi complex is organized as a stack of cisternae, with the cis-face receiving cargo from the ER and the trans-face or trans Golgi network (TGN) sorting cargo for export to their respective destinations [26]. The stacks are interconnected by tubular membranes into a continuous structure named the Golgi ribbon, which is a feature of the organelle in mammals. Within cells the Golgi is positioned close to the centrosomes, the primary microtubule-organizing center (MTOC) in dividing cells; vital for the maintenance of cell polarity, this center in turn modulates cell migration and neurite outgrowth [27]. The Golgi undergoes changes in morphology during various cellular processes such as cell cycle progression and stress responses [28]. The Golgi is highly dynamic, and undergoes disassembly during mitosis in early prophase and reassembly in telophase [29,30]. During mitosis in mammals, the Golgi ribbon is disassembled and partitioned into daughter cells. This disassembly also regulates mitotic progression [31].

### 2.2. The Golgi Stress Response

**Figure 2:**
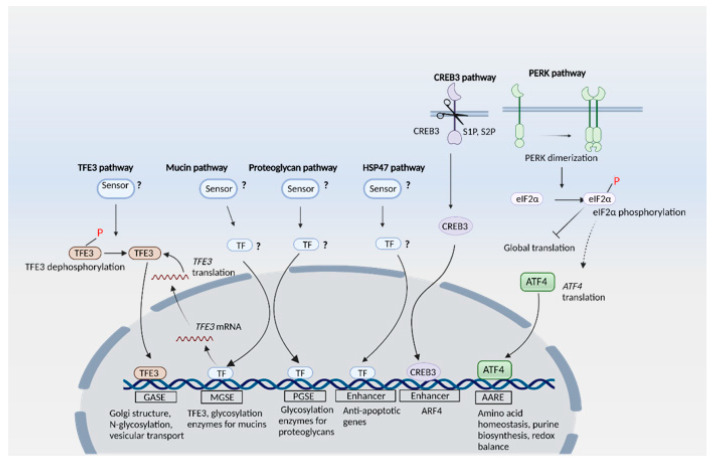
The Golgi stress response. Pathways involving the transcription factor E3 (TFE3), mucin, proteoglycan, heat shock protein 47 (HSP47), cAMP responsive element binding protein 3 (CREB3) and the protein kinase R (PKR)-like ER kinase (PERK) have been identified. The sensors for TFE3, mucin, proteoglycan and HSP47 have not yet been identified. The transcription factors for the mucin, proteoglycan and HSP47 pathways are also not well characterized. In the TFE3 pathway, dephosphorylation of TFE3 causes its nuclear translocation, where it activates transcription of its target genes by interacting with the Golgi apparatus stress response element (GASE) enhancer element. The mucin pathway, which is activated in response to insufficiency of mucin glycosylation, displays crosstalk with the TFE3 pathway. The mucin-type Golgi stress response element (MGSE) is present on the promoter of the *TFE3* gene as well. The proteoglycan pathway involves the ER-localized CREB3 (which functions as a sensor for Golgi stress) translocating from the ER to the Golgi to be cleaved by S1P and S2P proteases. The cytosolic region of the truncated CREB3 migrates to the nucleus and activates transcription of ARF4, leading to apoptosis. In the HSP47 pathway, expression of HSP47 (an ER chaperone involved in collagen folding and maturation) inhibits Golgi stress-induced apoptosis. The PERK pathway acts via the eIF2α/ATF4 axis, however, BiP/GRP78 is not induced when this pathway is activated by Golgi stress.

The Golgi plays central roles in glycosylation of proteins and harbors glycosyltransferases, glycosidases, and nucleotide sugar transporters, which orchestrate addition of various sugars that result in a mature glycan [32]. The cargo may also undergo several post-translational modifications including acetylation and phosphorylation, sulfation, methylation or proteolytic cleavage [33]. When the capacity of the Golgi is exceeded, it causes Golgi stress in a manner analogous to ER stress. In order to counter Golgi stress, cells mount signaling cascades which constitute the Golgi stress response [34]. Although the Golgi stress response is not as extensively characterized as ER stress, mounting evidence suggests the involvement of signaling cascades. Several signaling cascades have been reported with sensor proteins and effector proteins, which together elicit the Golgi stress response (Figure 2). These include the TFE3, proteoglycan, CREB3, PERK and HSP47 pathways [21,35,36].

#### 2.2.1. The TFE3 Pathway

The TFE3 pathway involves the activation of proteins responsible for the maintenance of Golgi functions. TFE3 targets include the Golgi structural proteins (GCP60, Giantin, and GM130) which maintain the structural integrity of the Golgi. GM130 (Golgin95) and Golgin-160 are cis-Golgi-localized harboring coiled-coils, and mediate stacking of Golgi cisternae and vesicular transport by serving as a vesicle tethering factor [37]. GM130 anchors adjacent stacks by interacting with Golgi reassembly and stacking protein of 65 kD (GRASP65) and the p115 protein through its C-terminal and N-domains, respectively [37]. GCP60 (also called acyl-CoA binding domain containing 3, or ACBD3) is a Golgi structural protein associated with Golgi integral membrane protein (Giantin). Overexpressing a dominant negative mutant of GCP60 triggers disassembly of the Golgi and blockage of protein transport from the ER to the Golgi [38]. Other targets of TFE3 include: *N*-glycosylation enzymes, such as ST3 beta-galactoside alpha-2,3-sialyltransferase 1 (SIAT4A/ST3GAL1), SIAT10, fucosyltransferase 1 (FUT1), Galactosylgalactosylxylosylprotein 3-beta-glucuronosyltransferase 2 (B3GAT2) and UDP-*N*-acetylhexosamine pyrophosphorylase-like protein 1 (UAP1L1); proteins involved in vesicular transport, such as Syntaxin 3A (STX3A), WD-repeat protein Interacting with phosphoinositide (WIPI)-1alpha (WIPI49/WIP1α) and RAB20 (Ras-related protein Rab-20); and Golgi proteases. Analysis of the promoters of ACBD3 and SIAT4A has revealed the presence of an enhancer element, termed the Golgi apparatus stress response element (GASE) [39]. Two transcription factors, TFE3 and MLX, were reported to bind GASE, which has a consensus sequence of ACGTGGC. Increasing the expression of TFE3 while increasing expression of MLX decreased transcription of genes with the GASE sequence. It has also been reported that Golgi stressors cause dephosphorylation of TFE3 at Ser108 and its nuclear translocation [40]. Similarly, MLX also translocates to the nucleus in response to Golgi stress [41]. Similar to ER stress, Golgi stress may trigger proteosomal degradation, which acts to restore Golgi homeostasis and organelle autoregulation. When Golgi stress persists, Golgi-apparatus related degradation (GARD) may ensue, analogous to ERAD associated with the ER. Golgi stress induces proteasomal degradation of GM130, which causes Golgi dispersal [42]. Other mechanisms proposed for regulation of Golgi tethering factors and morphology include caspase 3 mediated cleavage of GRASP-65 [43].

#### 2.2.2. The Proteoglycan Pathway

The proteoglycan (PG) pathway involves upregulation of glycosylation enzymes when PG glycosylation capacity in the Golgi is suboptimal. Genes encoding these glycosylation enzymes harbor the enhancer elements PGSE-A and PGSE-B (with consensus sequences CCGGGGCGGGGCG and TTTTACAATTGGTC, respectively) in their promoters.

#### 2.2.3. The CREB3 Pathway

In the CREB3 (ATF3) pathway, the ER-resident membrane protein CREB3 senses Golgi stress and translocates from the ER to the Golgi to be cleaved by S1P and S2P proteases. The truncated CREB3 derived from the cytosolic region moves to the nucleus and activates transcription of ARF4 to induce apoptosis [36].

#### 2.2.4. The HSP47 Pathway

The HSP47 pathway, on the other hand, acts to prevent apoptosis induced by Golgi stress by stimulating the expression of the ER-resident chaperone HSP47 to suppress Golgi stress-induced apoptosis [44].

#### 2.2.5. The Mucin Arm

The mucin arm, first proposed in 2019, is activated in response to inadequate mucin-type glycosylation in the Golgi. Mucins are high molecular weight, heavily glycosylated proteins produced by epithelial cells, which often form gel-like structures and are components of mucous. Expression of glycosylation enzymes for mucins such as GALNT5, GALNT8, and GALNT18 has been observed in this case. The mucin-type Golgi stress intersects with the TFE3 pathway as well, by inducing the expression and activation of TFE3. An enhancer element regulating transcriptional induction of TFE3 upon mucin-type Golgi stress was identified and designated as the mucin-type Golgi stress response element, with a consensus sequence ACTTCC (N9) TCCCCA [45].

#### 2.2.6. The PERK Pathway

The PERK pathway, which is also activated during ER stress, has additionally been identified as a pathway activated by the Golgi stressor monensin [21]. However, unlike ER stress, the ER-resident chaperone BiP/GRP78 is not induced during Golgi stress, suggesting that Golgi stress response is a distinct type of stress despite the phosphorylation of PERK in both types of stress response.

## 3. Golgi Stress Response and Redox Imbalance in Neurodegeneration: Focus on Huntington’s Disease

Accumulating evidence reveals that abnormalities in the structure and function of Golgi apparatus occur in neurodegenerative diseases including Alzheimer’s disease (AD), Amyotrophic lateral sclerosis (ALS), Parkinson’s disease (PD), Huntington’s disease (HD) and Creutzfeldt–Jakob disease [20,46,47,48,49,50,51]. The Golgi has also been reported to be fragmented during viral infection [52]. Depletion of the golgin GM130 has been reported to cause Golgi disruption, Purkinje neuron loss, and ataxia in mice [53]. Golgi fragmentation in dopaminergic neurons in the substantia nigra has been observed in Parkinson’s disease patients [54]. Early studies revealed that the Golgi apparatus may be fragmented in a population of neurons without neurofibrillary tangles (NFTs) [55]. In JNPL3 transgenic mice—which express the P301L mutant of tau, a component of NFTs and paired helical filaments (PHFs)—the Golgi complex was fragmented; however, mitochondria or other membranous organelles appeared normal, indicating that Golgi fragmentation is one of the earliest events that occur during pathogenesis, a finding which has been suggested by other laboratories as well [56,57,58]. Structural deformities in the Golgi complex were also linked to accumulation of phospho-tau in the P301S mouse model of AD [59]. Aging is a major risk factor for neurodegeneration; not surprisingly, increased Golgi fragmentation in neurons was observed with aging. GRASP65 and Golgin-84 were also diminished in the aging mouse brain [60].

In HD, we showed that elevated levels of ACBD3 occurred in cell culture and mouse models as well as human HD [20]. ACBD3 bound to the striatal protein ras homolog enriched in striatum (Rhes), which binds to mutant huntingtin (mHtt) and mediates cell death in HD [61]. More recently, we identified another arm of the Golgi stress signaling pathway in a striatal progenitor cell line model of HD. HD is a neurodegenerative disorder triggered by expansion of CAG repeats (encoding polyglutamine repeats) in the gene encoding huntingtin and which primarily affects the corpus striatum of the brain, manifesting as abnormal involuntary movements along with motor and cognitive deficits [1,2]. mHtt affects multiple cellular processes such as DNA replication and repair, transcription, translation, nucleocytoplasmic trafficking, mitochondrial function and proteostasis, to name a few [4,5,6,62,63,64,65,66].

### 3.1. Redox Imbalance and Cysteine Metabolism in HD

A hallmark of HD is increased oxidative stress. Oxidative stress occurs when the balance between prooxidant and antioxidant pathways tilts in favor of the former. However, oxidative stress has more recently been defined as a disruption of redox signaling pathways [67]. Elevated oxidative stress is at the heart of several neurodegenerative diseases, as well as other conditions [68,69]. Decreased levels and/or dysregulated metabolism of the antioxidants such as ascorbate (vitamin C), glutathione (GSH) and cysteine, and coenzyme Q10 (CoQ10) have been observed in HD and contribute to disease pathology [70,71,72]. Both biosynthesis and the uptake of cysteine or its oxidized form cystine are compromised in HD, causing elevated oxidative stress [5,73,74]. The activity of the neuronal cysteine transporter EAAT3/EAAC1 is reduced in HD due to decreased trafficking to the cell membrane [75]. Decreased expression of cystathionine γ-lyase (CSE) (the biosynthetic enzyme for cysteine) also occurs in HD as mHtt sequesters specificity protein 1 (SP1), the transcription factor for CSE during basal conditions [71,76,77]. CSE is also regulated by activating transcription factor 4 (ATF4) in response to stress. In HD-affected cells the induction of ATF4 is suboptimal, leading to decreased CSE expression and cysteine biosynthesis [5]. Cysteine is utilized in the biosynthesis of sulfur-containing molecules such as coenzyme A, taurine, lanthionine, homolanthionine, and cystamine [78]. It is also the substrate for production of the gaseous signaling molecule hydrogen sulfide (H_2_S) [79,80]. H_2_S signals by sulfhydration or persulfidation, a posttranslational modification which occurs on the –SH group of reactive cysteine residues, leading to formation of –SSH or persulfide groups [81]. Sulfhydration modulates the function of several proteins and signaling cascades, including response to inflammation, mitochondrial bioenergetics and stress responses [82,83]. Both cysteine metabolism and sulfhydration are altered in HD, which contributes to increased protein oxidation [5,71,84].

### 3.2. Golgi Stress Response and Links to Redox Homeostasis

ATF4, a master regulator of amino acid homeostasis and stress responses, is a central player in the integrated stress response [85,86,87]. It also regulates purine biosynthesis and regulates mTOR function [88]. Furthermore, ATF4 modulates the switch from synthesis of fetal hemoglobin to adult hemoglobin by stimulating transcription of BCL11A, a repressor of γ-globin synthesis [89]. ATF4 harbors a basic leucine-zipper (bZIP) domain and can either form homodimers or heterodimerize with other members of the bZIP family (FOS/JUN, ATF and CCAAT enhancer-binding protein (C/EBP) bZIP transcription factors) to control transcription [90,91]. Nuclear factor erythroid 2-related factor 2 (Nrf2), the master regulator of redox regulation, also forms heterodimers with ATF4 and stimulates the transcription of cytoprotective genes during oxidative stress [92]. ATF4 may have dual functions, modulating either cell survival or cell death, and excessive stimulation of ATF4 signaling may cause cell death [93]. The pro-survival or apoptotic function of ATF4 has been attributed in part to the identity of its heterodimerization partner and the physiologic context; the mechanisms are still being elucidated [94].

**Figure 3:**
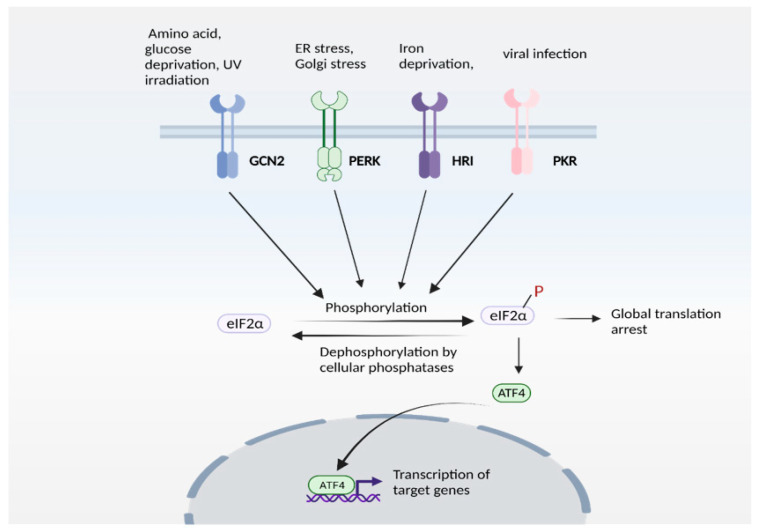
The integrated stress response (ISR). ISR is activated by several stress stimuli. In mammals, these are sensed by four kinases: general control non-depressible 2 (GCN2), protein kinase R protein kinase R (PKR)-like ER kinase (PERK), Heme-regulated eIF2α kinase (HRI) and double stranded RNA dependent protein kinase (PKR), each of which respond to a set of stimuli. These kinases undergo autophosphorylation and phosphorylate the eukaryotic initiation factor 2α (eIF2α), which inhibits its catalytic activity and its function of delivering the initiator tRNA to the ribosome, thereby arresting global translation. Under these conditions, only certain mRNAs (such as that encoding ATF4) are translated. ATF4 functions either as a homodimer or heterodimer to transactivate its target genes. The pathway is referred to as ISR because signaling mediated by diverse stress stimuli converge upon a common pathway (eIF2α/ATF4 axis).

**Figure 4:**
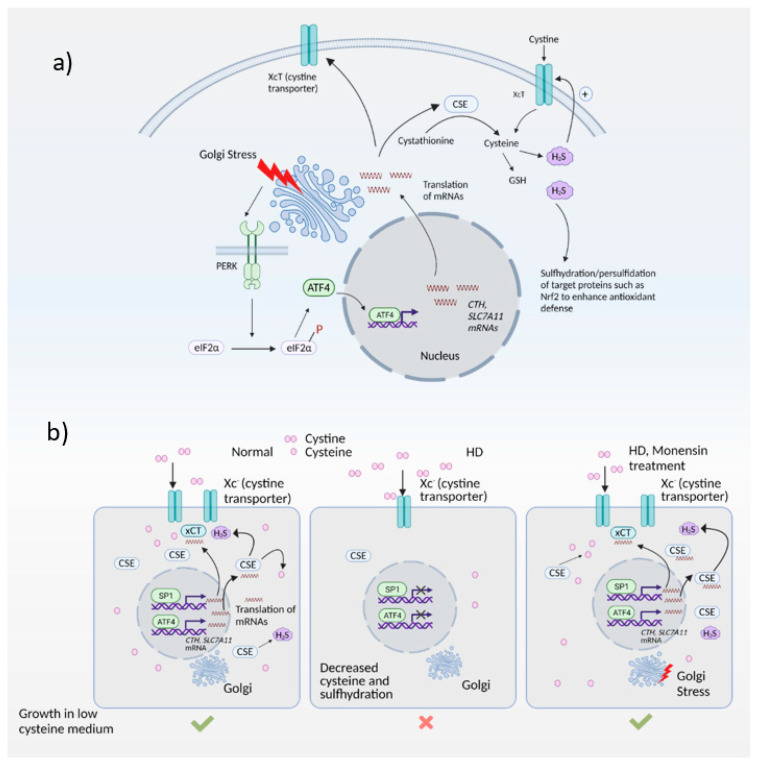
The Golgi stress response and its intersection with redox homeostasis in Huntington’s disease (HD). (**a**) Golgi stress response in normal cells. Golgi stress activates PERK, which phosphorylates eIF2α to inhibit general protein synthesis. Only mRNAs such as *ATF4* are translated. ATF4 regulates amino acid homeostasis and one of the genes induced by ATF4 is *CTH* (which encodes the biosynthetic enzyme for cysteine, also called CSE). CSE utilizes cysteine to produce the gaseous signaling molecule hydrogen sulfide (H_2_S). H_2_S signals by a post-translational modification termed sulfhydration/persulfidation and modulates the activity of target proteins. H_2_S stimulates cystine uptake by the cystine transporter, leading to increased cysteine levels in cells. ATF4 also regulates expression of SLC7A11 (xCT), a subunit of the cystine transporter, by activating its transcription through heterodimerization with Nrf2, a master regulator of redox homeostasis. (**b**) Harnessing the Golgi stress response to elicit cytoprotection in HD. Normal cells express CSE and ATF4 during basal conditions and during stress to produce cysteine. Cysteine is also imported into cells via the cystine transporter, Xc^-^. In HD, both basal expression of CSE (regulated by specificity protein1, SP1) as well as stress-induced expression of CSE and the xCT subunit of the cystine transporter by ATF4 are compromised, causing a cysteine deficit which leads to decreased H_2_S levels and sulfhydration. When cells are treated with monensin, a Golgi stressor, CSE is induced via the PERK/ATF4 pathway to increase cysteine and H_2_S levels and mediate cytoprotection.

Expression of ATF4 is regulated at the transcriptional as well as the translational level, and is a vital part of the integrated stress response [95,96]. The integrated stress response (Figure 3) is engaged in response to stress stimuli, including but not limited to amino acid and nutrient deprivation, ER stress, mitochondrial stress, iron dysregulation and viral infection. Four kinases, namely general control non-derepressible 2 (GCN2), PKR-like ER kinase (PERK) 19, double-stranded RNA-dependent protein kinase (PKR), and heme-regulated eIF2α kinase (HRI) sense the stress and phosphorylate the eukaryotic initiation 2 α (eIF2α), which abrogates its catalytic activity resulting in global translational arrest [85,97,98,99,100,101]. Under these conditions, only mRNAs responsible for maintenance of cell survival and essential signaling (such as ATF4) are translated. Recently, we showed that the Golgi stressors, monensin and nigericin, activated the integrated stress response by eliciting phosphorylation of PERK, resulting in translation of ATF4 and expression of its targets [21] (Figure 4). Among these targets were enzymes involved in cysteine biosynthesis and uptake, cystathionine γ-lyase (CSE) and SLC7A11, a subunit of the XcT transporter, which imports cystine, the oxidized form of cysteine (Figure 4a).

In the striatal progenitor cell culture models ST*Hdh*^*Q*7/*Q*7^ (Q7) and ST*Hdh*^1117/*Q*111^ (Q111), treatment with monensin rescued cell death associated with cysteine deprivation. Monensin increased the expression of ATF4 in the Q111 cells, which have compromised stress responses. We had shown previously that Q111 cells had decreased expression of CSE and thus could not grow in the absence of cysteine [5,71]. These cells were also compromised in their ability to upregulate ATF4, the transcription factor responsible for induction of CSE during cysteine deprivation. Treatment with monensin rescued growth in cysteine-free media and decreased oxidative stress in a manner dependent on PERK (Figure 4b). Monensin failed to upregulate ATF4 and CSE expression in cells deleted for PERK. Thus, Golgi stress engages the PERK pathway in HD cells. We further showed that mild Golgi stress can be harnessed to elicit cytoprotective effects [21].

## 4. Conclusions

Cells are outfitted with an array of defense mechanisms to counter stress. When exposed to stress stimuli, adaptive and cytoprotective pathways are engaged to restore balance. When the damage induced by stress cannot be resolved, apoptosis ensues. It is becoming increasingly clear that exposure to low-grade stress may precondition cells in a hermetic manner. Thus, mild stress such as low-grade Golgi stress, which does not cause toxicity, can up-regulate defense mechanisms to precondition cells to withstand future insults. Novel therapeutics which target the points of intersection between stress and adaptive responses may be beneficial in a wide variety of diseases.

## Abbreviations

ACBD3	Acyl CoA-Binding Domain Containing 3
ATF4	Activating Transcription Factor 4
BGAT2	Galactosyl Galactosyl Xylosyl Protein 3-β Glucuronosyltransferase 2
BiP	Binding Immunoglobulin Protein
CHOP	C/EBP Homologous Protein
CoQ10	Coenzyme Q10
CREB	cAMP Response Element Binding Protein 3
CSE	Cystathionine γ-lyase
GCN2	General Control non-Derepressible 2
eIF2α	Eukaryotic Translation Initiation Factor 2 Subunit-α
EAAT3/EAAC1	Excitatory Amino Acid Transporter 3
ER	Endoplasmic Reticulum
ERAD	ER-Associated Degradation
FUT1	Fucosyltransferase 1
GARD	Golgi-Apparatus Related Degradation
GASE	Golgi Apparatus Stress Response Element
GRASP65	Golgi Reassembly and Stacking Protein of 65 kD
HD	Huntington’s Disease
HRI	Heme-Regulated eIF2α Kinase
IRE1	Inositol-Requiring Enzyme 1
MTOC	Microtubule Organizing Center
NFTs	Neurofibrillary Tangles
Nrf2	Nuclear Factor Erythroid 2-Related Factor 2
PDI	Protein Disulfide Isomerase
PERK	Protein Kinase R Protein Kinase R (PKR)-Like ER Kinase
PG	Proteoglycan
PHF	Paired Helical Filament
Rab20	Ras-Related Protein Rab-20
Rhes	Ras Homolog Enriched in Striatum
RIDD	Regulated IRE1-Dependent Decay
S1P	Site 1 Protease
S2P	Site 2 Protease
SIAT4A/ST3GAL1ST3	β-Galactoside Alpha-2,3-Sialyltransferase 1
SP1	Specificity Protein 1
STX3	Syntaxin 3A
TFE3	Transcription Factor E3
TGN	Trans Golgi Network
UAP1L1	UDP-*N*-Acetylhexosamine Pyrophosphorylase-Like Protein 1
UPR	Unfolded Protein Response
WIPI49/WIP1α	WD-Repeat Protein Interacting with PhosphoInosides 1α
XBP1	X-box Binding Protein 1
